# Improving Breast Cancer Control via the Use of Community Health Workers in South Africa: A Critical Review

**DOI:** 10.1155/2011/150423

**Published:** 2010-09-26

**Authors:** Brianna M. Wadler, Christine M. Judge, Marianne Prout, Jennifer D. Allen, Alan C. Geller

**Affiliations:** ^1^Division of Public Health Practice, Harvard School of Public Health, 677 Huntington Avenue, Landmark 3rd Floor East, Boston, MA 02115, USA; ^2^Department of Epidemiology, Boston University School of Public Health, 715 Albany St, Talbot Building, Boston, MA 02118, USA; ^3^Center for Community-Based Research, Cantor Center for Nursing Research and Patient Care Services, Dana-Farber Cancer Institute, 44 Binney Street, Boston, MA 02115, USA

## Abstract

Breast cancer is a growing concern in low- and middle-income countries (LMCs). We explore community health worker (CHW) programs and describe their potential use in LMCs. We use South Africa as an example of how CHWs could improve access to breast health care because of its middle-income status, existing cancer centers, and history of CHW programs. CHWs could assume three main roles along the cancer control continuum: health education, screening, and patient navigation. By raising awareness about breast cancer through education, women are more likely to undergo screening. Many more women can be screened resulting in earlier-stage disease if CHWs are trained to perform clinical breast exams. As patient navigators, CHWs can guide women through the screening and treatment process. It is suggested that these roles be combined within existing CHW programs to maximize resources and improve breast cancer outcomes in LMCs.

## 1. Introduction

While breast cancer has long been recognized as a major public health burden in high-income countries, the majority of cases actually occur in low- and middle-income countries (LMCs), and it is expected that incidence rates will rise most rapidly in these locations [1]. The relative burden of mortality is also higher in less developed countries than in more developed countries, as indicated by higher mortality : incidence ratios (0.44 versus 0.29, resp.) [2]. Current global initiatives focus on developing and implementing resource-appropriate guidelines and strategies to improve breast health care and breast cancer outcomes in LMCs [3–5]. 

Common challenges cited for resource-poor countries include limited health care infrastructure [6], later stages at diagnosis, and competing health care priorities [7]. The purpose of this paper is to examine the potential for community health worker (CHW) programs to improve access to breast health resources in LMCs. To this end, we briefly review the effectiveness of CHW programs in LMCs and identify key components of a CHW-based breast health program. We focus on South Africa as an example country to assess the feasibility of such a program. South Africa represents a middle-income country, according to its World Bank classification [8], has a growing cancer control infrastructure including many of the cancer centers in Africa, with well-trained oncologists and radiologists [9, 10], has historical experience with community-based health worker programs [11, 12], and has a higher breast cancer mortality : incidence ratio compared to the world standard (Table 1), indicating lower survival from breast cancer [2, 13]. Importantly, it also enjoys advocacy from its First Lady, Her Excellency Madam Tobeka Stacie Madiba-Zuma, who serves as Vice Chairperson of the newly-formed Forum of African First Ladies Against Breast and Cervical Cancer [14]. Still, barriers to breast care persist. Many women do not have access to the information and screening necessary to prolong survival, as evidenced by the high mortality : incidence ratio in South Africa. These factors illustrate both the need and potential assets for a successful CHW breast health program.

Data from South Africa's National Cancer Registry (NCR) show breast cancer as the leading cancer among women [15]. South African women have a 1 in 29 lifetime risk of developing breast cancer, with an age-standardized incidence rate of 30.6 per 100,000 population. These rates vary by race group, with Black women having the lowest (16.3) and White women the highest (69.4) rates of breast cancer diagnosis. The NCR is a pathology-based, rather than a population-based “registry” “therefore” these statistics underestimate cancer incidence in South Africa. These statistics belie marked disparities in stages of cancers at diagnosis, survival rates and overall breast cancer in South Africa [16]. However, the indication of disparities along racial lines adds urgency to the call for expanded access to breast cancer screening, diagnostic services, and treatment, particularly through community-based approaches.

**Figure 1:**
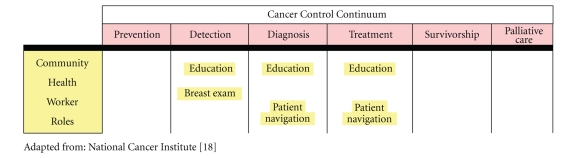
The Cancer Control Continuum and Suggested Roles of CHWs in LMCs.

The cancer control continuum is a commonly used public health framework that describes the various stages at which potential programs or interventions can be developed to improve cancer outcomes for population groups (Figure 1) [17]. For CHWs to intercede with the objective of reducing stage of breast cancer at diagnosis and increasing survival, target areas along the cancer continuum should be early detection, diagnosis, and treatment. Below we present evidence to develop a program model for a successful breast health program focusing on these areas along the continuum.

## 2. Community Health Workers

### 2.1. Definition

Community health workers are “members of the communities where they work, should be selected by the communities, should be answerable to the communities for their activities, should be supported by the health system but not necessarily a part of it” [18, p. 6]. In the literature, the most frequently identified roles of CHWs are health education, health services provision, and patient navigation and support. Health education is one of the most common roles of CHWs in all types of settings [11, 12, 19–21]. Patient navigation—helping patients find their ways through health systems to ensure timely screening, diagnosis, and treatment—is also mentioned frequently as a role for CHWs (e.g., [12]) and has been successfully employed in the field of cancer [22].

Because they work in their own communities, CHWs presumably have a shared life experience and understand the sociocultural context in which health services are received and health behaviors occur [11, 19–21]. Many other terms are used to describe CHWs, including lay health workers, village health workers, or “community care workers,” specifically in South Africa [23].

### 2.2. Community Context and Roles

CHWs have an extensive history of action in communities throughout the world, but especially in LMCs. One important aspect of CHW programs is the necessity of integration with the community [12, 21]. As highlighted in the WHO report, “more important is an acknowledgement that the definition of CHWs must respond to local societal and cultural norms and customs to ensure community acceptance and ownership” [21, page v]. In this same report, the authors note that the CHW literature is “unanimous” in saying that the communities need to assume ownership for CHW programs to work successfully and that such programs work best when the community has a strong investment in the program [21].

In many areas, CHWs are the only source of health services [21]. These services include, for example, malaria treatment, as indicated in studies about Burkina Faso [24], and antiretroviral medication administration [12]. In Haiti, researchers from Partners in Health have concluded that CHWs have strengthened the health system by providing services to rural communities that would otherwise not be reached [25]. In a review of CHWs in Africa, authors argue that in order to expand health services on the African continent, CHWs are a necessity [11]. Lehmann and Sanders [21] note that the shortage of health workers is significant in places like sub-Saharan Africa (see [26]) and that these gaps in services could be filled by CHWs.

### 2.3. Evidence

Several reviews have examined the impact of CHW programs throughout the world across a range of health outcomes. In a recent review [27] of 82 randomized controlled trials (RCTs) of lay health workers (33% of the studies took place in LMCs), most demonstrated improved health behaviors or health outcomes as a result of CHW interventions. In one RCT in low socioeconomic communities in South Africa, CHWs were trained to give parenting support and guidance to new mothers [28]. Mothers who received this intervention were significantly more sensitive in their infant interactions at both 6 and 12 months (*P* < .05), and more infants had securely attached to mothers at 18 months (75% versus 63%, *P* < .05). 

A descriptive and historical review from the WHO included approximately 250 citations about CHWs. Authors concluded that “robust evidence” supports the positive impact CHWs can have on health outcomes [21, page 26]. In another review of CHW interventions, a case management model for pneumonia led to a 24% reduction in overall mortality under age five across several countries [29].

Apart from the RCTs referenced above, a large body of evidence about CHW effectiveness is available from observational and descriptive studies [21], including two studies in South Africa highlighting the importance of CHWs. In a review of a developing national CHW program, authors argued that CHWs were seen as an important element in a “cross-sectoral” response by the South African government [30]. In a longitudinal study of antiretroviral therapy in Free State Province, patients visited by CHWs at 6 months had significantly increased probability of having CD4 counts higher than 200 cells/*μ*L at 1 year (*P* < .05). Those visited at 12 months, compared to patients without CHWs, were significantly more likely to be considered treatment successes at 24 months. They argued that CHWs were part of the untapped community resource available to provide “chronic disease care” [31, page 1184]. 

A qualitative review across all provinces of South Africa recognized the importance of CHWs' work [12]. Authors found that CHWs increasingly provided health services, such as antiretroviral medication administration, in addition to health promoting activities. They helped to expand health services available, especially in impoverished areas of the country, and assisted patients in navigating the health system [12, page 3]. In another qualitative study, CHWs in KwaZulu-Natal excelled at identifying community problems because of their connection to community. However, they also quickly named the obstacles to providing services in these locations, raising the need for regular monitoring and support for CHW programs [32]. A cross-sectional study in Cape Town found that women contacted by a CHW were more likely to return for a cervical cancer screening visit. Loss-to-follow-up was reduced from 21% to 6% for 6-month visits and reduced by half for 24-month visits [33]. 

In summary, there is widespread evidence that CHW programs can be an effective part of improving community health, particularly in limited-resource areas of the world. Community health worker approaches are proving beneficial in the areas of immunization uptake, breastfeeding, tuberculosis (TB) treatment, and child morbidity and mortality [27]. Given the long history of the use of lay health workers in Africa [11] and lessons learned about the successes and challenges of creating effective CHW programs in South Africa and other LMCs [12, 23], the capacity to expand to breast cancer appears to be feasible. The use of CHWs should be considered as a key resource-appropriate strategy to bring culturally appropriate breast health services to women.

## 3. Suggested Implementation

### 3.1. CHW Roles in Breast Cancer

Based on the literature, CHWs in South Africa could assume three primary functions: health education, health service provision (i.e., breast exam), and patient navigation. Health education has been a premier role for CHWs in multiple settings. Integrating breast cancer education into existing health education would be vitally important because knowledge is an essential starting place in establishing the need for breast cancer screening [34]. Awareness varies widely among women [35, 36]; in some settings, breast cancer is stigmatized [37] or considered contagious [6]. Particularly in rural areas of South Africa, breast cancer may be understood as a curse or poison sent by a sorcerer [38]. CHWs, because of their grounding in the community, are uniquely prepared to understand and acknowledge local beliefs or “myths” and provide information about causes of the disease, which could help to destigmatize breast cancer. Women are more likely to engage with CHWs from their own communities, who understand and respect their beliefs and concerns and have earned their trust. Education is more likely to be successful when in such cases. 

In addition to education, CHWs would also provide clinical breast exams (CBEs) as a form of early detection. Although mammography is the standard screening for breast cancer in high-income countries, population-based screening is not feasible in many LMCs due to high costs of the required equipment and personnel. One study in India, for example, found that mammography was not as cost-effective as CBE [39]. CBE is a low-cost method of screening women for breast cancer in lower resource areas, such as parts of South Africa. CBE has been successfully taught to lay health workers in other settings [40] and used on a large scale, similar to CHW cervical cancer screening implemented in rural Alaska, U.S.A. [41]. Recent research supports the use of role play as a method of health skills training in limited-resource settings [42]. CHWs could be taught breast self-exam and then CBE by practicing on other CHWs in a “train the trainer” method.

The goal of CHW-delivered CBE would be to downstage presentation of breast cancer in LMCs [1, 43]. Currently the majority of breast cancers found in Africa are in stages III and IV [44, 45]. Trials of CBE in Cairo [46] and Mumbai [40], where cancer is also found at late stages, have shown that CBE as a primary screening tool can be provided by lay persons as a sustainable form of early detection. In Malaysia, use of CBE was shown to increase downstaging [47].

 CHWs can also serve as patient navigators through the continuum of breast cancer screening, diagnosis, and treatment. Already seen as links or “bridges” to local health systems [48], CHWs can be highly effective in assisting patients to maximize their access to existing systems, thereby reducing potential delays in care [49]. Barriers to care in LMCs can include traveling to health centers, lack of affordable services, and cultural challenges with seeking care [22]. For example, CHWs have successfully acted as “treatment buddies” in HIV treatment in South Africa [31]. As navigators, they provide emotional and logistic support, which can be the crucial element in enabling patients to access any form of care [50]. In one study, rural African-American women, who had lay health advisors contacting them as an intervention, reported an 11-percentage-point increase in mammography compared to women who did not receive the intervention [51]. A recent study from Ethiopia illustrates the inefficient multistep health care journey of many breast cancer patients, supporting the need for streamlined patient navigation as a way to save resources and time in LMCs [49].

### 3.2. Program Integration

Although many current CHW programs are disease-specific [21], convincing arguments exist for integrating several health services within one CHW program's domain. One economic exercise presented the potential benefits of bundling services for various health concerns together in LMCs [52]. The authors found that packaging services together in this way would expand the possibilities for reaching populations in LMCs because of reduced cost. More recently, in treatment of HIV/AIDS and TB in Africa, authors have argued that CHWs fit best into an entire community health team, operating most effectively in a generalized, instead of disease-specific, way [53]. Screening for breast and cervical cancer could be fit into regular primary care visits conducted by health workers, such as CHWs [54]. For example, Knaul et al. have suggested that breast cancer screening could be integrated into existing reproductive health programs [55].

A cost-effectiveness simulation of screening in India, a lower middle-income country [8], indicated that CBE would have the greatest impact with women ages 40–60 compared with ages 50–70 [56]. Furthermore, Miller argues, based on early findings from The Cairo Breast Screening Trial, that all women ages 40–69 should receive CBE [46]. Although women with a family history of breast cancer are often targeted in other countries, this approach does not seem feasible in LMCs because of poor record keeping and accuracy of the reporting of breast cancer. 

### 3.3. Community Involvement

Across all discussions of CHWs, the importance of community involvement is repeated regularly. Such involvement can help to ensure that sociocultural norms and customs are recognized and respected. In the past, South African communities with a vested interest in CHW programs benefited most from the programs [12]. These communities participated in identifying their own needs and potential solutions, which increased the success of CHW programs [20]. The South African government notes the importance of encouraging community members to define their own needs [23].

Once community members are invested in a CHW program, they must be involved in nominating candidates to become CHWs. Doing so increases the likelihood that CHW candidates are respected members of the community who will be effective communicators, educators, and service providers [20]. Some communities in South Africa have also incorporated traditional healers into CHW programs, which can enhance programmatic success [12]. 

### 3.4. Program Sustainability

The longer a CHW program is established in a community, the more successful it can become [12]. Maintaining CHW programs requires the ongoing support of the community, in addition to ongoing resources and training. Periodic “refresher” trainings for CHWs have been especially effective in keeping programs productive and useful to their respective communities [11].

Often in community—nongovernment organization (NGO)—government partnerships, confusion ensues over responsibilities for funding and program management. According to the South African Department of Health [12], governments ideally provide funding through NGOs, which in turn employ CHWs. The most recent draft document from the Departments of Health and Social Development describes this approach using the general partnerships within South Africa (e.g., [23]). However, changes in policy and leadership can lead to changes in funding, leaving NGOs and CHWs without the essential resources and supplies to continue their work. Because of this challenge, CHW programs that build capacity within communities are more sustainable and rely less on outside funding from NGOs or parts of the government. The South African government notes the importance of increasing community capacity [23]. For example, the concept of “training up” workers so that existing CHWs take on more skills, such as CBE, builds capacity within the community. This example has also been described as “task shifting,” in which each level of health worker takes on additional skills, such as a general nurse performing oncology duties [6].

## 4. Discussion

### 4.1. Benefits of a Community Health Worker Breast Cancer Screening Program

How would such a CHW program benefit women and their families? By educating women about breast health, women in the community would be better poised to seek essential services. Because of misconceptions about breast cancer and fears about how it could affect their families, many women do not seek care until it is too late [35]. Women who are diagnosed when their cancer is in earlier stages and connect with CHWs can successfully access and receive treatment in a timely manner and have better chances of survival. 

A CHW program for breast cancer as described here must address problems specific to screening in LMCs. One of the predominant problems in screening for breast cancer is a shortage of trained personnel who can deliver breast health services [6], aggravated in Africa by the “brain drain,” in which talented clinicians leave for better situations outside Africa. The clinical breast exam has been used successfully for screening in other studies, and CHWs have been able to learn and successfully administer the exam [40]. With CHWs performing routine screenings, medical staff can be freed for more skilled tasks. For patient navigation, CHWs should be trusted members of the women's communities and be able to link them to post-screening care after a positive finding. They should also offer guidance to help women understand their diagnoses and courses of treatment. Patients who work with CHWs are more likely to adhere to follow-up treatment because they have a better understanding of the health system and the course of their treatment. This enhanced understanding saves clinical time and resources and can lead to better outcomes for patients [22]. 

### 4.2. Challenges

There are many challenges to breast cancer control in LMCs such as South Africa. The use of CHWs to perform CBE is fairly new in the field of breast cancer care, and more evidence-based studies or program evaluations are needed to detail methods that successfully incorporate this component. First, in other LMCs facing demanding health concerns such as access to clean water and infectious disease control, some may question the priority of the time and expense needed to initiate a successful program. Second, the lack of reliable population-based cancer data for most of Africa is a barrier to understanding the full extent of the breast cancer burden. The limited data available suggest that breast cancer incidence rates may peak at younger ages [16]. Third, sub-optimal resources can hamper the development of new programs in low- and middle-income countries. Lack of food, income, transportation, and other conditions of poverty limit the ability of people to access health programs and services. To successfully maintain a CHW screening program, several components must be in place. We contend that breast cancer screening can be woven into existing infrastructure already prominent in many middle-income countries. South Africa is presented here as an example because, as an upper middle-income country, mammography is available in some areas and the country has a history of CHW programs. The present recommendations are not suitable for all countries or all communities but are intended as a conceptual model for how services may be expanded.

### 4.3. Future Research

Future research should test the proposed program in different settings. First, rigorous evaluations must be implemented to assess efficacy of such programs, with an emphasis on community members' involvement in assessing program effectiveness. Important research questions include: What are the best methods for preparing CHWs for their role? How much initial and re-training is needed? Are such programs acceptable to the community (including women and those who may have power over their decisions and actions)? For information on longer-term programmatic effects, follow-up studies of women participating in the programs could measure women's attitudes, knowledge, and practices over time as well as track changes in external determinants of health that influence the feasibility of women participating in breast health care. Depending on the variability of conditions that could influence CHW programs, randomized controlled trials may be necessary. A key resource in ongoing research and implementation is the Breast Health Global Initiative (BHGI), which recently opened the BHGI Learning Laboratory in Kumasi, Ghana and is training a new set of breast cancer health practitioners [57].

The magnitude of lives lost to breast cancer in South Africa and throughout low- and middle-income countries is unacceptable, and in large part, preventable. By building on existing infrastructure, utilizing lower-cost health service options, such as CHWs, and engaging in partnerships with affected communities, there is great potential to reduce the burden of breast cancer. 

## Figures and Tables

**Table 1:** Female breast cancer incidence rates, mortality rates, and mortality : incidence ratios for selected countries and the world, by World Bank income classification^*a*, *b*^.

Country name	Region	Incidence rate^cf^	Mortality rate^df^	Mortality to incidence ratio^e^
	
World	World	37.4	13.2	35.3

*Middle incom-upper* ^b^				
**Botswana**	**Africa**	**33.4**	**25.0**	**74.9**
Brazil	Latin America	46.0	14.1	30.7
Colombia	Latin America	30.3	12.5	41.3
**Gabon**	**Africa**	**18.2**	**13.1**	**72.0**
Lebanon	Middle East	52.5	23.4	44.6
Malaysia	East Asia	30.8	13.5	43.8
Mexico	Latin America	26.4	10.5	39.8
**Namibia**	**Gabon**	**24.7**	**18.8**	**76.1**
Panama	Latin America	29.0	12.0	41.4
Peru	Latin America	35.1	14.0	39.9
Romania	Europe	44.3	16.7	37.7
***South Africa***	***Africa***	***35.0***	***16.4***	***46.9***
Turkey	Europe	22.0	9.7	44.1

*Middle income lower* ^b^				
**Bolivia**	**Latin America**	**24.7**	**11.6**	**47.0**
Honduras	Latin America	25.9	12.1	46.7
Jordan	Middle East	33.0	14.6	44.2
**Nigeria**	**Africa**	**31.2**	**21.9**	**70.2**
**Sudan**	**Africa**	**22.5**	**16.6**	**73.8**

*Low income* ^b^				
**Mozambique**	**Africa**	**3.9**	**2.8**	**71.8**
**Tajikistan**	**Europe**	**13.2**	**6.2**	**47.0**
**Uganda**	**Africa**	**18.3**	**13.4**	**73.2**
Vietnam	East Asia	16.2	7.1	43.8
Yemen	Middle East	35.1	15.6	44.4
**Zimbabwe**	**Africa**	**19.0**	**14.1**	**74.2**

*High income* ^b^				
Australia		83.2	18.4	22.1
Italy	Europe	74.4	18.9	25.4
Japan	Asia	32.7	8.3	25.4
United States	North America	101.1	19.0	18.8

*N* *o* *t* *e* *s*: (a) bold type indicate higher mortality: incidence ratios than South Africa, (b) income classification based on World Bank list of economies (July 2009) [8], (c) incidence rate (IR): number of new cases of breast cancer per 100,000, age-standardized to the world population (ASR), (d) mortality rate (MR): Number of deaths due to breast cancer per 100,000 (ASR), (e) Mortality to Incidence Ratio = MR/IR, (f) IR and MR from GLOBOCAN 2002 [13].
